# Pilot case control evaluation of artificial intelligence assisted orthodontic monitoring and pediatric patient perception

**DOI:** 10.1038/s41598-025-24395-9

**Published:** 2025-11-18

**Authors:** Ana Martínez Gil-Ortega, Patricia Cintora-López, Luis Miguel Pérez Rodríguez, María José Viñas, Juan Manuel Aragoneses, Patricia Arrieta-Blanco, Andrea Martín-Vacas, Marta Macarena Paz-Cortés

**Affiliations:** 1 Centro Odontológico de Innovación y Especialidades Avanzadas, Universidad Alfonso X El Sabio, Madrid, 28037 Spain; 2 Facultad de Odontología, Universidad Alfonso X El Sabio, Villanueva de la Cañada, 28691 Spain; 3 Department of Dental Research, Federico Henriquez y Carvajal University, Avenida Isabel Aguiar, 100, 10106 Herrera, Santo Domingo Oeste Dominican Republic

**Keywords:** Orthodontics, Telemedicine, Orthodontic appliances, Removable, Dentistry, Pediatric dentistry, Dentistry, Paediatrics

## Abstract

Artificial Intelligence (AI) has become a key tool in the modernization of the healthcare industry, aiding dentists in performing their work more efficiently and effectively. The aim of this study was to evaluate orthodontic monitoring and patient perception using the AI-assisted Dental Monitoring software in children. The study was designed as a randomized controlled case-control study, including retrospective data collection through patients’ medical records. Dental Monitoring application enables patients to scan or capture images of their dentition using a smartphone, allowing orthodontists to remotely check treatment progress. Children aged between 7- and 12-years undergoing treatment with Invisalign First were invited to take part. They were classified into two groups based on orthodontic monitoring method: conventional methods (control group) and Dental Monitoring (DM) software. Outcomes included demographic variables, Angle molar classification, initial number of aligners, treatment duration in months, number of refinements, number of aligners in the first refinement, number of aligners in the second refinement, number of appointments, number of emergencies, and patient perception. Data were analyzed for statistical significance, applying a 95% confidence level. The study included a total of 39 patients (20 in the DM group and 19 in the control group). Both groups were homogeneous in terms of age, sex, and malocclusion. No significant differences were observed between the two groups, except for the number of appointments, which was significantly lower in the DM group compared to the control group (*p* < 0.001). Regarding children’s perception, 85% found scanning to be either very easy or easy, and 100% of the patients were satisfied or very satisfied with their communication with the orthodontist. Moreover, 100% of patients were satisfied or very satisfied with the DM application, and 85% would recommend the experience. The group monitored using DM showed a significant reduction in the number of appointments compared to the control group, with no significant differences in treatment duration, number of refinements, or number of aligners per refinement. Children reported a highly favorable perception of orthodontic monitoring with DM.

## Introduction

Artificial Intelligence (AI) has become a key tool in the modernization of the healthcare industry, enabling professionals and organizations within the sector to perform their work more efficiently and effectively^1^. Telemedicine, or remote healthcare, is also an established reality in contemporary dental practice^2–5^. This remote monitoring approach offers multiple advantages for orthodontists, including a reduction in the need for in-person visits, which benefits not only dental professionals but also patients. A second advantage of remote monitoring is the early detection of issues such as poor oral hygiene^6^, aligner misfit, appliance breakage, or insufficient patient compliance, ultimately contributing to a reduction in overall treatment duration^7^. Additionally, as reported by various authors, remote monitoring helps better supervision of patient adherence and ensures the proper progression of orthodontic treatment. Lastly, it serves as a valuable communication tool between clinicians and patients^5,7–9^.

At the forefront of tele-orthodontic technology is the *Dental Monitoring* (DM) application, which enables patients to scan or capture images of their dentition using a smartphone, allowing orthodontists to remotely check treatment progress^1,10^. Researchers such as Morris et al.^11^, Moylan et al.^12^, Kurakose et al.^13^, and Homsi et al.^14^ have studied and confirmed the clinical accuracy of DM in tracking tooth movement across distinct types of orthodontic appliances.

The DM application is a software system including a mobile application accessible to patients, an algorithm-based processing system, and a web platform through which orthodontists receive updates on their patients’ treatment progress^1,4,7,9^. This technology enables patients to accurately capture the current state of their dentition using AI, smartphones, and specialized cheek retractors^1,8^. Patients receive in-clinic instruction on how to use the application and perform the required scans, with the frequency of these scans decided by the orthodontist^2^. As orthodontic treatment progresses, DM allows patients to update their records, communicate with their orthodontist, and reduce the number of in-person visits to the dental clinic. Meanwhile, orthodontists can asynchronously monitor treatment progress, helping to prevent unforeseen issues for patients and facilitating the scheduling of necessary follow-up appointments to ensure best orthodontic outcomes.

Several studies in the existing literature have proved the effectiveness of monitoring through the DM application^2–5,8,9,14–21^. Additionally, numerous studies have explored the use of DM for other applications within dentistry. Morris et al.^11^ utilized the application to assess the three-dimensional accuracy of digital models generated from videos and images captured with the software. Moylan et al.^12^ evaluated the system’s reliability and precision in measuring linear tooth movements. Furthermore, linear movements have been studied in the context of accelerating orthodontic treatment^22^. Impellizzeri et al.^10^ confirmed the application’s effectiveness in patients treated with self-ligating fixed multibracket appliances. Other researchers, such as Snider et al.^19,20^ and Sangalli et al.^6^, have highlighted the utility of DM in supporting proper oral hygiene during orthodontic treatment. Additionally, authors like Sangalli et al.^23^ and Surovková et al.^24^ have reported on the use of DM during the retention phase, proving its benefits in remotely monitoring patients and reducing in-office visits, an advantage for both patients and clinicians. Lastly, several studies have investigated patient and clinician perceptions of the application^8,16,21,25–28^. These surveys consistently conclude that DM is well-accepted among users, holds significant potential, and is considered a valuable tool for both patients and orthodontic professionals.

Digital technologies are increasingly being integrated into orthodontic practice to enhance diagnostic accuracy and treatment planning, resulting in improved efficiency and precision^29,30^. The adoption of CAD/CAM systems, intraoral scanners, and advanced image processing softwares has enabled comprehensive digitalization of patient records and workflows^29–34^. Among these innovations, digital bracket bonding stands out as a key advancement, allowing for virtual bracket placement based on three-dimensional models generated via CAD/CAM technology. This approach significantly improves bonding accuracy by eliminating operator-dependent manual errors during the procedure^35–42^. AI, often referred to as the “Fourth Industrial Revolution”^43–45^, has seen widespread implementation across medical and dental disciplines. In orthodontics, AI-driven applications have been developed to analyse skeletal relationships, assess facial aesthetics, and predict post-pubertal mandibular growth. A growing body of research supports the validity of AI in diagnostic processes, treatment planning, monitoring, and therapeutic decision-making, with outcomes comparable to those achieved by experienced clinicians^43,46^. Consequently, the integration of AI contributes to a more precise and personalized approach to patient care.

Currently, no published studies in scientific literature have compared pediatric patients treated with clear aligners with and without DM. Given that children stand for a considerable proportion of orthodontic patients and that there is an increasing demand for more aesthetic treatment options, we considered it proper to conduct a study assessing the usefulness of this AI-based application in younger patients. The null hypothesis stated that no differences would be- observed between the two groups, while the alternative hypothesis is based on a better patient perception using AI-based application to monitor the orthodontic treatment. The aim of this study was to evaluate orthodontic monitoring and patient perception using AI-assisted *Dental Monitoring* software in children treated with *Invisalign First*, compared to a control group.

## Materials and methods

### Study design and ethical concerns

For the conduction of this longitudinal study, prior approval was obtained from the Alfonso X El Sabio Institutional Ethics Committee (code 2024_5/270, accepted on May 22nd, 2024). The study was designed as randomized controlled case-control research, with retrospective data collection through patients’ medical records. The research design and method followed STROBE guidelines^47^. As the participants were under 12 years of age, parental informed consent was required before the study began. Furthermore, all procedures adhered strictly to current regulations concerning personal data protection and the guarantee of digital rights.

### Participants and study sample

A set of inclusion and exclusion criteria was established to refine the selected sample. The inclusion criteria included children aged 7 to 12 years undergoing orthodontic treatment with clear aligners (*Invisalign First*), with skeletal class I (ANB angle between 0 and 4 degrees) or mild class II (ANB angle between 4 and 6 degrees), no dental agenesis, and treatment not requiring extractions. Exclusion criteria included patients with skeletal Class III (ANB angle < 0 degree) or moderate to severe skeletal Class II (ANB angle > 6 degrees), children undergoing combined treatment with fixed appliances, or those with preformed metal crowns. Skeletal class was assessed according to Spanish reference values^48^, as norm values for ANB are 2 ± 2 degrees. A moderate range of sagittal problems (mild class II) was included, but children with extreme class II (> 2 standard deviations from published norms) were excluded.

Patients were recruited through non-probabilistic consecutive case sampling between May and December 2024, inviting those attending orthodontic check-ups who voluntarily agreed to take part. Due to the pilot design, sample size was not calculated. Parents were informed about the study and invited to take part voluntarily. Upon their consent, an Informed Consent form was provided to parents or legal guardians. Each enrolled patient was assigned a numerical code, which was also used to name the aligners used by each participant.

### Outcomes

Demographic variables (age and gender) were collected in order to analyze sample distribution. Orthodontic outcomes, such as Angle molar class^49^, skeletal malocclusion classification (ANB angle assessment from Steiner cephalometry) were recorded.

Orthodontic monitoring^8,9,17,18^ was evaluated in numerical terms according to the following variables^1^: initial number of aligners^2^, treatment duration in months^3^, number of refinements^4^, number of aligners in the first refinement^5^, number of aligners in the second refinement^6^, number of appointments, and^7^ number of emergency visits. Upon completion of the study, an anonymous satisfaction survey was conducted with the children, adapted from the studies published by Hansa^8,16^ and translated into Spanish by a native bilingual researcher. The survey consisted of 11 questions, with responses based on a 5-point Likert scale.

### Study procedure

The treatment was conducted by a single, experienced orthodontist, who was previously calibrated and holds the necessary accreditation to use *Invisalign* aligner treatments (Align Technology, Santa Clara, California) and the *Dental Monitoring* application (version 2.00 to 7.34 according to application updates during orthodontic treatment, https://dentalmonitoring.com/, Paris, France) in a private dental clinic.

Prior to the commencement of orthodontic treatment, patients underwent an occlusal study, including a panoramic radiograph (Owandy i-max 3D ceph XPRO), a lateral cephalometric radiograph (Owandy i-max 3D ceph XPRO), an extraoral and intraoral photographic series (Olympus OM-D E-M5 camera with Olympus ED 60 mm f/2.8 macro lens), and an intraoral scan (iTero Element, Align Technology). All records were taken at the same dental clinic, under artificial lighting and consistent environmental conditions (light, temperature, and humidity). The photographic series and intraoral scan were uploaded to the *Invisalign* website to develop the treatment plan and fabricate the aligners. All patients were treated with *Invisalign* First clear aligners, custom-made for each patient’s teeth using ClinCheck software (version Pro 6.0, https://www.invisalign.com/provider/align-digital-platform/clincheck, Align Technology, Santa Clara, California), which plans the required dental movements.

On the first day of treatment, patients were instructed on how to download and install the DM software on their smartphones. Additionally, the patients were provided with instructions on how to perform the necessary scans through the app to ensure proper monitoring of their treatment progress.

Patients who chose to use the AI tool for orthodontic monitoring were instructed, at the start of treatment, to change aligners every 7 days (with monitoring via DM) and to attend in-office check-ups every 8–10 weeks. With each aligner change, the app uses AI to analyze the fit of the new aligner, providing the patient with a “GO” if the aligner change is proper, or a “NO-GO” if the patient needs to keep the same aligner for a longer period. Simultaneously, the orthodontist receives this information about the patient’s progress, deciding whether the patient can move to the next aligner or if there are any misalignments or hygiene issues. The orthodontist can modify the AI’s response if desired^8^. On the other hand, patients who were not monitored with the DM application are also instructed to change their aligners every 7 days and attend in-office check-ups every 4–5 weeks.

The patients included in the study were followed up until the completion of their orthodontic treatment to complete the satisfaction survey, and the data related to their orthodontic treatment were obtained from their medical records.

### Data analysis

All data were collected using Microsoft Excel (Microsoft 365). Statistical analysis of the data was performed with the SPSS statistical software (version 29.0, Armonk, NY, USA). A 95% confidence level (*p* ≤ 0.05) and asymptotic or bilateral significance were used in all analyses. For the descriptive statistics of quantitative variables, the Kolmogorov-Smirnov and Shapiro-Wilk normality tests were applied. For qualitative variables, Fisher’s Exact Test or the Chi-square test were used, and the non-parametric Mann-Whitney test was used to compare a quantitative variable between the two groups. The effect of study group, age, gender and ANB on the number of clinical appointments was studied with a multiple linear regression analysis. Besides, the effect of demographic outcomes (gender and age) on the significant Items of the satisfaction survey were analysed with an ordinal regression method. Likert scale scores were also assessed using symmetry and kurtosis statistics.

## Results

The final sample consisted of 39 participants (22 males and 17 females) with a mean age of 9.36 ± 1.22 years. The study groups were composed of 19 subjects in the control group and 20 in the DM group. Molar class I stood for 50% in the study group and 42.10% in the control group, with no subjects with molar class III included. The groups were homogeneous in terms of gender and molar class distribution (χ² *p* = 0.855 and *p* = 0.621, respectively). A description of quantitative variables was conducted (Table 1). Data analysis revealed that the variables Age in the study group, ANB, number of refinements, number of aligners in the first refinement in the control group, number of aligners in the second refinement, and number of emergency visits did not meet normality criteria (Kolmogorov-Smirnov and Shapiro-Wilk tests, *p* < 0.05 for all comparisons). The remaining variables followed a normal distribution.

**Table 1:** Descriptive data and normality test results of the studied variables.

	Control			Dental monitoring			Total	U Mann-Whitney
	Descriptive statistics	Normality test		Descriptive statistics	Normality test		Descriptive statistics	
	Mean ± SD	KS Sig.*	SW Sig.	Mean ± SD	KS Sig.	SW Sig.	Mean ± SD	Sig.
Age	9 ± 1.41	0.101	0.136	9.7 ± 0.92	0.009*	0.017*	9.36 ± 1.22	0.113
ANB	3.79 ± 1.23	0.023*	0.041*	3.95 ± 1.19	0.020*	0.039*	3.87 ± 1.20	0.687
Number of aligners	46.84 ± 12.74	0.200	0.612	51 ± 12.08	0.200	0.275	48.97 ± 12.42	0.270
Months of treatment	24.63 ± 6.18	0.200	0.793	25.65 ± 4.74	0.200	0.145	25.15 ± 5.44	0.531
Number of refinements	1.47 ± 0.51	0.001*	0.001*	1.60 ± 0.60	0.001*	0.001*	1.54 ± 0.55	0.607
Number of aligners (1st refinement)	35.37 ± 15.24	0.004*	0.012*	32.45 ± 15.47	0.063	0.072	33.87 ± 15.23	0.478
Number of aligners (2nd refinement)	10.74 ± 13.08	0.001*	0.001*	18.55 ± 17.95	0.002*	0.003*	14.74 ± 16.06	0.175
Number of appointments	31.58 ± 8.07	0.200	0.769	21.65 ± 4.51	0.200	0.213	26.49 ± 8.14	< 0.001*
Number of emergency visits	1.47 ± 1.5	0.035*	0.013*	0.75 ± 0.97	0.001*	0.001*	1.10 ± 1.29	0.149

*KS* Kolmogorov-Smirnov test, *SW* Shapiro-Wilk test.

*P value. Statistical significance *p* < 0.05.

**Fig. 1:**
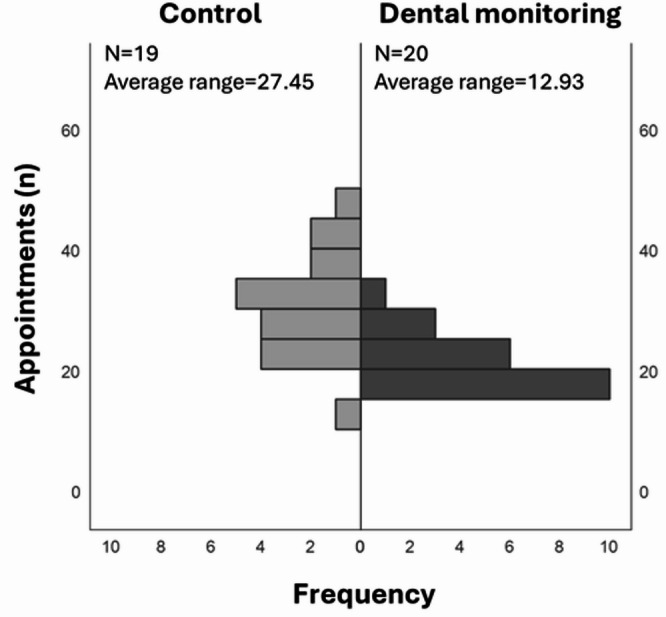
Significant differences in the number of appointments between the control group and the DM group.

Differences in the analyzed variables between the study groups were assessed. Significant differences were shown exclusively in the variable “number of appointments” (Mann-Whitney *p* < 0.001), with no significant differences seen in the remaining variables (Mann-Whitney *p* > 0.05 for all comparisons). The data show that the control group had a mean of 31.58 ± 8.07 appointments, while the study group needed fewer appointments (21.65 ± 4.51), with a mean difference of 9.93 appointments (a reduction in 68.56% of the appointments) (Fig. 1).

A multiple linear regression analysis was conducted to evaluate the influence of study group, age, gender, and ANB on the number of clinical appointments (Table 2). The overall model was statistically significant (F(4,34) = 8.128, *p* < 0.001), accounting for 48.9% of the variance in appointment frequency (R² = 0.489). Study group emerged as the strongest predictor (β = 0.643, *p* < 0.001), indicating that patients belonging to control group required significantly more appointments. ANB also demonstrated a significant positive association (β = 0.356, *p* = 0.013), suggesting that increased ANB values are correlated with a higher number of clinical visits. Neither age nor gender were significant predictors, implying that these demographic factors do not substantially impact appointment frequency in this cohort.

**Table 2:** Multiple linear regression analysis evaluating the influence of study group, age, gender and ANB on the number of clinical appointments.

Model	Non-standardized coefficients		Standardized coefficients	t	Sig.
	B	Standard error	β		
(Constant)	40.746	90.734	–	0.488	0.629
Group	100.336	20.063	0.643	50.011	< 0.001*
Age	-0.044	0.874	−0.007	-0.051	0.960
Gender	-10.667	20.160	−0.103	-0.772	0.446
ANB	20.426	0.930	0.356	20.608	0.013*

*P value. Statistical significance *p* < 0.05.

**Fig. 2:**
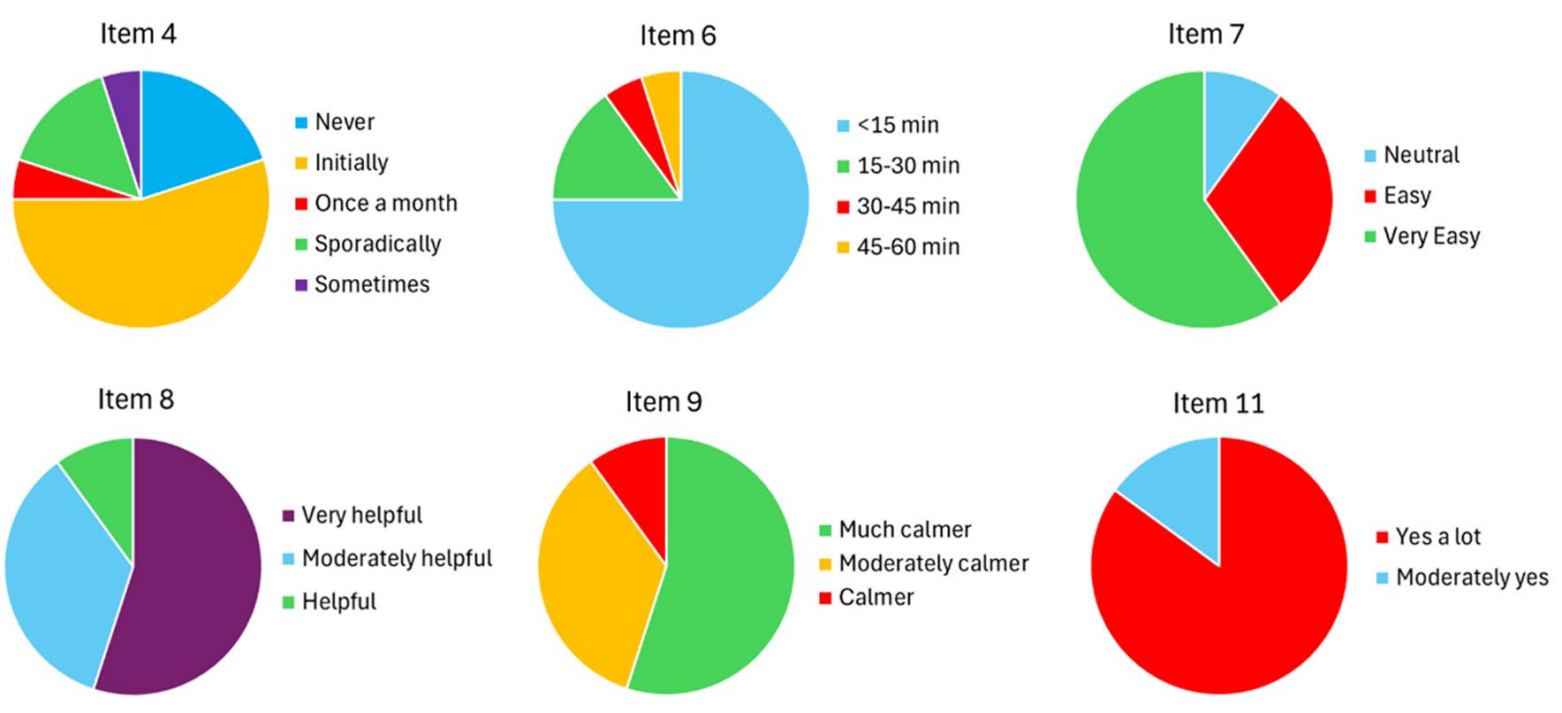
Survey’s items with statistically significant differences in the responses.

Patient perception was analyzed, revealing significant differences in responses to Items 4, 6, 7, 8, 9, and 11 (Table 3; Fig. 2). However, no significant differences were seen in the remaining items evaluated (χ² *p* > 0.05 for all comparisons). Consequently, we found that there are no significant differences among respondents on scanning difficulty, patient ability, satisfaction with communication levels, overall satisfaction, or perceived therapeutic benefits in the evaluation of DM. Nevertheless, 50% of respondents described the application as very easy to use, while 45% reported good ability to perform scans regularly and on time. Satisfaction with communication with the orthodontist ranged from moderate (41.1%) to high (40%), and overall satisfaction was rated as good (45%) or very good (55%). All respondents expressed a positive opinion about the benefits of DM, perceiving it as beneficial (60%) or highly beneficial (40%). However, these findings do not indicate significant differences.

**Table 3:** Description and significance values of the satisfaction questionnaire filled by DM users in the study sample.

Item/Question		Responses	*N* (%)	χ^2^ *P* value
1	How difficult is it to take scans using DM?	Very easy	10 (50%)	0.157
		Easy	7 (35%)	
		Indifferent	3 (15%)	
2	How would you rate your ability to perform weekly scans regularly and on time?	Poor	1 (5%)	0.094
		Normal	5 (25%)	
		Good	9 (45%)	
		Excellent	5 (25%)	
3	Are you satisfied with the level of communication with the orthodontist DM?	Satisfied	12 (60%)	0.371
		Very satisfied	8 (40%)	
4	How often are your scans rejected and require retaking?	Never	4 (20%)	0.002*
		Initially	11 (55%)	
		Once a month	1 (5%)	
		Sporadically	3 (15%)	
		Sometimes	1 (5%)	
5	What has your overall level of satisfaction with DM?	Satisfied	9 (45%)	0.655
		Very satisfied	11 (55%)	
6	What is your travel time to the dental clinic?	< 15 min	15 (75%)	< 0.001*
		15–30 min	3 (15%)	
		30–45 min	1 (5%)	
		45–60 min	1 (5%)	
7	How easy is it to use the DM application?	Neutral	2 (10%)	0.022*
		Easy	6 (30%)	
		Very easy	12 (60%)	
8	Do you think dental monitoring through DM has helped you stay committed to your dental treatment?	Very helpful	11 (55%)	0.047*
		Moderately helpful	7 (35%)	
		Helpful	2 (10%)	
9	Do you feel calmer knowing that you are constantly monitored by your doctor throughout your treatment via the application?	Much calmer	11 (55%)	0.047*
		Moderately calmer	7 (35%)	
		Calmer	2 (10%)	
10	How beneficial has DM been in your treatment experience?	Beneficial	12 (60%)	0.371
		Very beneficial	8 (40%)	
11	Would you recommend this experience to other patients?	Yes, a lot	17 (85%)	0.002*
		Moderately yes	3 (15%)	

*P value. Statistical significance *p* < 0.05.

Regarding Item 4, it was found that scans frequently (55%) needed to be retaken after being initially rejected (χ² *p* = 0.002). Concerning travel time to the clinic, 90% of participants reported a travel time of less than 15 min (75%) or between 15 and 30 min (15%) (χ² *p* < 0.001). The majority (60%) of participants rated the use of DM as very easy, followed by easy (30%) (χ² *p* = 0.022). Additionally, 55% of respondents said that DM had significantly helped them stay more committed to their treatment and feel much calmer on their orthodontic care (χ² *p* = 0.047 for both cases). Lastly, all participants showed that they would recommend DM, with 85% describing it as highly recommendable (χ² *p* = 0.002).

The effect of gender and age on the survey items that yielded statistically significant results was examined using an ordinal regression model. The analysis revealed that demographic variables did not significantly influence responses to Items 4 (*p* = 0.133), 6 (*p* = 0.134), 8 (*p* = 0.093), 9 (*p* = 0.186), and 11 (*p* = 0.236). However, although the model was statistically significant for Item 7 (χ² = 6.595, *p* = 0.037, Nagelkerke R² = 0.337), neither age nor gender showed a significant effect on the response categories. Notably, female gender exhibited a trend (β = 2.312) toward higher scores regarding the ease of use of DM (*p* = 0.06).

Table 4 summarizes descriptive statistics for the survey items. Items 5, 7, and 10 showed high mean values (≥ 4.40), while Items 6, 8, 9, and 11 had notably low scores (≤ 1.55). Skewness values varied, with strong positive skew in Items 6 and 11, indicating clustering at lower response categories. Negative skew was observed in Items 2, 5, and 7, suggesting a tendency toward higher ratings. Kurtosis values revealed leptokurtic distributions in Items 6 and 11, while Items 3, 5, and 10 were platykurtic, indicating flatter distributions.

**Table 4:** Descriptive statistics of the satisfaction questionnaire filled by DM users in the study sample.

Item	Mean ± SD^+^	95% confidence interval		Asymmetry ± SD	Kurtosis ± SD
		Lower	Upper		
1	1.65 ± 0.167	1.30	1.61	0.69 ± 0.51	-0.76 ± 0.99
2	3.90 ± 0.19	3.50	4.29	-0.36 ± 0.51	-0.30 ± 0.99
3	4.40 ± 0.11	4.16	4.64	0.44 ± 0.51	-2.02 ± 0.99
4	2.30 ± 0.25	1.77	2.83	1.05 ± 0.51	0.47 ± 0.99
5	4.55 ± 0.11	4.31	4.79	-0.22 ± 0.51	-2.18 ± 0.99
6	1.40 ± 0.18	1.02	1.78	2.26 ± 0.51	4.90 ± 0.99
7	4.50 ± 0.15	4.18	4.82	-1.08 ± 0.51	0.08 ± 0.99
8	1.55 ± 0.15	1.23	1.87	0.89 ± 0.51	-0.24 ± 0.99
9	1.55 ± 0.15	1.23	1.87	0.89 ± 0.51	-0.24 ± 0.99
10	4.40 ± 0.11	4.16	4.64	0.44 ± 0.51	-2.02 ± 0.99
11	1.15 ± 0.08	0.98	1.32	2.12 ± 0.51	2.78 ± 0.99

*SD* Standard deviation.

## Discussion

Tele-dentistry offers multiple benefits for patients, as it reduces visits and emergencies in the dental office, shortening waiting times and providing higher quality oral care. Besides, virtual consultations enable the possibility of examining more patients in a single day, shortening waiting times at dental office^50^. Tele-dentistry has also been proposed as a supportive method for patient treatment and monitoring. DM is an application that can be used for remote orthodontic monitoring in both children and adults. Our goal was to conduct a study comparing the effectiveness of the application in a sample of pediatric patients treated with aligners, monitored with the DM application, in comparison with a control group (without tele-dentistry). While there are studies evaluating its use in adult orthodontics, as discussed below, no similar studies have been found in pediatric populations like ours.

Regarding studies that use DM in pediatric populations, Caruso et al.^7^ published a case of an 11-year-old child treated with clear aligners, showing the successful follow-up conducted with DM during the COVID-19 pandemic. Other studies with sample sizes ranging from 12 to 30 subjects (ages 10–17) evaluate the reliability of DM scans by measuring intercanine and intermolar widths, comparing them with direct measurements and plaster models. These studies found that reliability is adequate, as the differences were not significant^12,13^. However, they do not assess monitoring during orthodontic treatment.

In contrast, there are studies in adult populations that evaluate two study groups with DM monitoring^8,9,16,18^. In all cases, except for the study by Hansa et al.^16^ (which had more males than females), the study groups were composed of subjects with similar age, gender, and malocclusion type to avoid inclusion biases. Gender equity is important, as it was concluded that females are more compliant than males on appointments and monitoring. In our study, the sample consisted of a homogeneous group of children in terms of age and gender.

About the sample size in adult studies, the total samples range from 56 to 159 subjects, divided into study and control groups^8,9,16,18^. In all these studies, the monitoring of DM in invisible orthodontics is evaluated. In our case, we evaluated 39 children. As for the number of first aligners, it is important to have homogeneity in the sample to properly analyze the number of refinements and later aligners, as well as the number of appointments. In our study, the number of first aligners was homogeneous, as in other studies^8,9,18^.

We analyzed whether tele-dentistry influenced the refinement after active treatment. About the number of refinements, we did not find significant differences between the study and control groups, which aligns with Hansa et al.^8^, who reports a homogeneous number of refinements, aligners in refinements, and time until the first refinement. However, in another study by the same author^9^, there was a trend toward a reduction in the number of refinements and a significantly shorter time until the first refinement was found in the group monitored with DM.

We also evaluated the treatment duration, finding no significant differences between the group with virtual monitoring and the control group. These results align with those of Hansa et al.^8,9^, where no statistically significant differences were found about treatment duration in months between the study group and the control group. In contrast, the study conducted by Lam et al.^18^ found a significantly longer treatment duration in the group monitored with DM (a difference of 1.9 months).

The number of emergency visits was also not different between the evaluated groups, which is consistent with earlier studies^9^. Regarding the number of orthodontic review or control appointments, we found a significant decrease in the number of visits in the DM group independently of age or gender (a reduction of 68.56% of the visits, with a mean difference of 9.93 visits), which aligns with the existing literature in this area of study^8,9,16,18^. Also, the number of appointments was positively correlated to ANB. The reduction in the number of visits reported by previous authors is lower than that obtained in this study, with a reduction of 23-33.1% in the number of visits (1.68–3.5 visits)^8,9^. This decrease in the number of visits is beneficial for both orthodontists, in terms of efficiency, and for the patient, as it reduces the inconvenience of attending the clinic and the transportation costs. However, it is important to note that both DM and Scan Box have a monthly cost per patient, which increases the expenses for the dental clinic. In an article about a case^22^ treated with invisible aligners and corticotomies, it was determined that the use of DM allowed aligner changes to be made every fewer day, accelerating the treatment and detecting misalignments earlier, thereby reducing the number of visits to the dental office. In another study conducted with 35 patients receiving fixed multibracket orthodontics, a significant reduction in the number of visits in the DM group was also seen, as well as the cost of the treatment for the clinic^10^.

The experience and satisfaction of patients using DM were assessed through a survey conducted within the dental clinic, adapted from previous studies published by Hansa^8,16^. This survey utilized a 5-point Likert scale (from 1, very dissatisfied, to 5, very satisfied), whereas alternative studies^18^ have employed a visual-analog scale (VAS) for evaluation. The findings from our study showed an elevated level of overall satisfaction among the patients, with 45% reporting satisfaction and 55% indicating they were very satisfied. These results surpass those previously reported in adult populations, where 71.9% of participants expressed either satisfaction or high satisfaction^8^. Regarding the ease of performing the scans, the majority (85%) of patients reported that it was easy or very easy, which is notably higher than the findings from earlier studies (68.8%)^8^. Additionally, only 5% of patients in our study reported poor scanning skills with DM, compared to 9% in the study by Hansa et al.^8^. While earlier studies reported that 41% of scans were never rejected, the rate in our study was lower at 20%, although 55% of scans were rejected only at the first stage. Significantly, 55% of participants reported feeling considerably more at ease knowing they were being remotely monitored. Caruso et al.^7^ evaluated two clinical cases (aged 11 and 57 years) treated with aligners and monitored using DM. They concluded that the use of the application enhanced communication between the dentist and patient, leading to successful monitoring outcomes.

Our results suggest that DM is an application well-accepted by patients and efficient in terms of reducing clinic time. It allows us to conduct treatments in the same manner as with non-monitored patients, as traditionally done, but with the advantage of extending the time between patient visits, thereby increasing the efficiency of our time in the clinic. Furthermore, DM is a technologically advanced tool that can enhance the patient’s perception of treatment quality and accuracy. The decision to use this technological application will ultimately depend on individual considerations, including the number of patients treated in the clinic, available space, costs, scheduling organization, and protocols.

DM has also been used for other dental purposes, such as monitoring oral hygiene^6,19,20^ and orthodontic retention visits^23,24^. Additionally, the accuracy of 3D scans with DM has been evaluated in studies^11,12^, proving good reliability and precision in reproducing dental arches.

The study conducted has some limitations that should be considered when interpreting the data. Firstly, dealing with children and adolescents can sometimes compromise the treatment, either due to limitations in cooperation, communication barriers, ability or skill to monitor and scan their teeth, or lack of commitment to the treatment. Moreover, developmental factors, such as a child’s ability to follow instructions, could affect DM’s efficacy compared to adult populations. Additionally, the retrospective nature of data collection using patient records may introduce a slight bias (incomplete documentation, reliance on medical record, variability in record-keeping practices), as it depends on the correct completion of these records. Furthermore, the low sample size due to the pilot aspect of the research could limit the generalizability of the results. Stratifing by age in younger and older children could have benefited the study, strengthening the clinical applicability of the findings. Other limitation of this study was the use of multiple bivariate analyses, which may increase the risk of Type I statistical errors due to repeated testing. Additionally, although χ² tests were employed to explore associations within the group, this method is not optimal for Likert-type scales, which are ordinal in nature. However, given the intragroup design and absence of comparison groups, alternative analytical approaches were not feasible, potentially limiting the depth and precision of data interpretation.

One of the main strengths of this study lies in its focus on a paediatric and adolescent cohort, a population underrepresented in previous research on remote orthodontic monitoring. By evaluating the use of Dental Monitoring in younger patients, the study provides novel insights into usability, engagement, and satisfaction in a group with distinct behavioural and developmental characteristics. The inclusion of real-world data enhances ecological validity, and the use of validated survey items adapted from prior studies ensures consistency with existing literature. On the other hand, children’s greater understanding of the digital world eases the use of mobile devices and applications by the pediatric population, who find digital monitoring more appealing than conventional methods. Additionally, this study has the significant advantage of being the first with a case-control design in a pediatric population, allowing for a comparative evaluation of variables rather than an empirical one.

Further larger multicentric prospective trials are needed to confirm the found outcomes (ej. reduced clinical appointments, high satisfaction) across diverse populations. Controlling several variables as patient understanding of DM tool operation, child maturity or manual dexterity could be interesting, in order to individualize orthodontic planification. Besides, future studies should prioritize prospective, real-time data collection to minimize biases and risks. The research would benefit from exploring cost-effectiveness or long-term outcomes, as post-treatment stability, retention phases and duration.

## Conclusion

Derived from the results obtained, there is a beneficial effect of orthodontic monitoring with DM in children, with a significant reduction in the number of visits, without increasing the treatment time or the number of aligners needed. The high patient satisfaction indicates that DM is perceived as easy to use, helpful, recommended, and beneficial, making patients feel more at ease as they are continuously monitored virtually.

## Data Availability

The raw data used and/or analyzed during the current study is available from the corresponding author on reasonable request.

## Abbreviations

χ^2^: Chi Square test
AI: Artificial intelligence
DM: Dental monitoring
KS: Kolmogorov–Smirnov test
SD: Standard deviation
Sig.: Signification
SW: Shapiro-Wilk test
